# Enhanced CO_2_ Adsorption by Nitrogen-Doped Graphene Oxide Sheets (N-GOs) Prepared by Employing Polymeric Precursors

**DOI:** 10.3390/ma11040578

**Published:** 2018-04-10

**Authors:** Abdulaziz Ali Alghamdi, Abdullah Fhead Alshahrani, Nezar H. Khdary, Fahad A. Alharthi, Hussain Ali Alattas, Syed Farooq Adil

**Affiliations:** 1 Department of Chemistry, College of Science, King Saud University, P.O. 2455, Riyadh 11451, Saudi Arabia; a.t.alshahrani@hotmail.com (A.F.A.); fharthi@ksu.edu.sa (F.A.A.); Attashussain@gmail.com (H.A.A.); 2 King Abdulaziz City for Science and Technology (KACST) P.O. Box 6086, Riyadh 11442, Saudi Arabia; nkhdary@kacst.edu.sa

**Keywords:** nitrogen-doped graphene oxide, polypyrrole, polyaniline, CO_2_, adsorption studies

## Abstract

Nitrogen-doped graphene oxide sheets (N-GOs) are prepared by employing N-containing polymers such as polypyrrole, polyaniline, and copolymer (polypyrrole-polyaniline) doped with acids such as HCl, H_2_SO_4_, and C_6_H_5_-SO_3_-K, which are activated using different concentrations of KOH and carbonized at 650 °C; characterized using SEM, TEM, BET, TGA-DSC, XRD, and XPS; and employed for the removal of environmental pollutant CO_2_. The porosity of the N-GOs obtained were found to be in the range 1–3.5 nm when the KOH employed was in the ratio of 1:4, and the XRD confirmed the formation of the layered like structure. However, when the KOH employed was in the ratio of 1:2, the pore diameter was found to be in the range of 50–200 nm. The SEM and TEM analysis reveal the porosity and sheet-like structure of the products obtained. The nitrogen-doped graphene oxide sheets (N-GOs) prepared by employing polypyrrole doped with C_6_H_5_-SO_3_-K were found to possess a high surface area of 2870 m^2^/g. The N-GOs displayed excellent CO_2_ capture property with the N-GOs; PPy/Ar-1 displayed ~1.36 mmol/g. The precursor employed, the dopant used, and the activation process were found to affect the adsorption property of the N-GOs obtained. The preparation procedure is simple and favourable for the synthesis of N-GOs for their application as adsorbents in greenhouse gas removal and capture.

## 1. Introduction

CO_2_ emission is a growing problem, which is mainly caused due to the burning of fossil fuel for the production of energy, which is used to drive the present day factories and automobiles. According to the report published by International Monetary Fund (IMF), there are 35.9 billion tonnes of carbon dioxide emissions worldwide, which is a dangerously alarming level, effecting human lives and ecological systems. Apart from its impact on the environment, studies have revealed that CO_2_ emissions have an impact on the gross domestic product (GDP) as well [1].

In order to overcome this problem, efforts are being made throughout the world to curb pollutions by replacing fossil fuels with other sources of sustainable energy. Apart from this, there are efforts such as afforestation and reforestation for the consumption of CO_2_ from the environment [2]. Other than this, there are attempts being made to enable adsorption of CO_2_ into materials, which is also known as CO_2_ capture and sequestration (CCS) technology, which can be employed further for the generation of electricity [3,4,5,6,7].

One of the most commonly used technique is entrapment of CO_2_, which is carried out by adsorption techniques using various adsorbents such as MOFs [8], mesoporous material such as MgO [9], metal decorated phosphorene [10], silica mesospheres [11,12], nanostructrual copolymer, and ionic liquid [13], etc. Carbon-based material such as carbon nanotubes [14], graphene [15], carbon spheres [16], activated carbon, and activated carbon fibers [17] has been extensively used for the entrapment CO_2_. Among these, graphene and graphene oxide has been extensively used for this study due to its superior surface area among the various carbon compounds used [18,19]. These were further modified by impregnating it with various metal salts such as Fe_3_O_4_ [20], Mn_3_O_4_ [21], Cu [22], Ca [23], and polymeric additives such as PEI [24] to improve the capture and entrapment of CO_2_. Further modification is carried out using nitrogen doping of the graphene sheets, wherein the nitrogen-doped graphene was employed for the capture of CO_2_, and it was found to possess better adsorption and selectivity towards CO_2_ [25].

Nitrogen-doped graphene is synthesized by employing various methods such as graphene exposed to ammonia vapors at elevated temperatures, graphene, graphene mixed with melamine as a source of nitrogen doping on the graphene sheets, the hydrazine steaming process, and the arc discharge method [26,27,28,29]. However, based on the information obtained from the previous literature, it can be understood that it is the need of the hour to find easier methods for the synthesis of this important material.

Hence, in continuation of our work on the synthesis of graphene and graphene-based composites for various applications [30,31,32], in this study, we prepared the N-doped graphene oxide sheets by employing ‘N’-containing polymers such as polypyrole, polyaniline, and copolymers (polypyrrole-anlline) doped with acids such as HCl, H_2_SO_4_, and C_6_H_5_-SO_3_-K, which were activated using different concentrations of KOH. The as-prepared carbonaceous materials were characterized using SEM, TEM, BET, XRD and XPS and tested for CO_2_ capture property.

## 2. Materials and Methods

All materials were obtained from commercial sources such as potassium salt benzene sulphonic acid (97%) and Sulphuric acid (98%) were procured from BDH, Poole, UK, while the polymeric precursors, i.e., monomers, such as Pyrrole (98%) and Aniline (99%), were procured from Alfa Aesar, Karlsruhe, Germany. Hydrochloric acid (~36%), Nitric acid (68%), and Methanol (99.5%) were procured from Fisher Chemical, Loughborough, UK. Ammonium persulphate (98%) and Pottassium hydroxide pellets (85%) were procured from Alfa Aesar, Karlsruhe, Germany. Solvents such as Acetone (99.5%) were procured from Panreac, Barcelona, Spain, and Isopropanol (+99%) was produced from WiNLAB, Middlesex, UK. All solvents were used as received without further purification.

The surface areas, pore size, and pore volumes of the prepared nitrogen-doped graphene oxide (N-GOs) were determined through nitrogen adsorption at 77 K, using Automated gas sorption system Micromeritics analyzer (Gemini VII, 2390 Surface Area and Porosity, Micromeritics, Norcross, GA, USA). Before analysis, each sample was degassed at 150 °C (under N_2_ flow) for 1 h to eliminate moisture and gasses. The specific surface area (SBET) was calculated by Brunauer, Emmett, and Teller (BET) method using adsorption isotherm in the range of 0.05 ≤ p/p_o_ ≤ 0.30. The average pore width and micro-pore volume were measured by Dubinin-Radushkevich (DR) equation from the N_2_ adsorption isotherm. (DR) equation is expressed by:

WWo=exp [−BT2 βln2 (pop)]

in which

W = volume of the pores that has been filled at p/p_o_ (cm^3^/g)
W_o_ = total volume of the micropore system (cm^3^/g)
β = structural constant related to the width of the Gaussian pore distribution (K^−2^)
T = temperature at which the isotherm has been taken (K)
B = similarity constant, depending solely on the adsorbate (-)
p_o_/p = inverse of the relative pressure of the adsorbate (-)

The total pore volume was estimated by Barrett, Joyner, and Halenda (BJH) model from the quantity of N_2_ adsorbed at relative pressure (p/p_o_) of 0.99 by the software of the instrument. The meso-pore volume was calculated by subtracting the micro-pore volume from the total pore volume:

V_Meso_ = V_t_ − V_Mic_

in which V_Meso_ is the mesopore volume, V_t_ is the total pore volume, and V_Mic_ is the micropore volume.

Scanning electron microscopy (SEM) images were obtained using JSM-6380-LA (JEOL, Tokyo, Japan), which was used for morphology analysis showing the surface texture, pore structure, and pore distribution of the prepared samples. Transmission electron microscopy (TEM) images were recorded on JEM-1011, transmission electron microscope (JEOL, Tokyo, Japan).

Carbon dioxide uptake and the heat of adsorption of the treated nitrogen-doped graphene oxide sheets (N-GOs) were measured by Thermo Gravimetric Analyser (TGA)/Differential Scanning Calorimetry (DSC) SDT-Q600, (TA instruments, New Castle, DE, USA) in the temperature range of 25–1000 °C at a heating rate of 10 °C min^−1^, using 99.9999% purity Carbon dioxide, additionally purified by a molecular sieve filter. Before analysis, each sample of 5–15 mg was cleaned up at 25–120 °C to eliminate moisture and gasses (under He flow), and the samples’ heating rate was 10 °C min^−1^, using isotherm 120 °C for 30 min. The carbon dioxide uptake experiments were performed at 50 °C, using uptake isotherm at 50 °C, and the flow rate of carbon dioxide was maintained at 100 mL/min for 30 min. The results are calculated as mg/g measurement, which is converted to mmol/g and presented as such.

### 2.1. Experimental

#### 2.1.1. Polymer Preparation

##### Polyaniline (PANI) and Polypyrrole (PPy)

0.04 mol of monomer (aniline, pyrrole) was taken in a round bottomed flask along with 0.06 mol of desired dopants (HCl, H_2_SO_4_, C_6_H_5_-SO_3_-K), and the mixture of reaction was stirred for 30 min at 0–5 °C. In another beaker, 13.7 g Ammonium persulphate (APS) was dissolved in 30 mL water and stirred in beaker for 30 min at 0–5 °C, which was added to the previous reaction mixture while stirring at 0–5 °C; the solid product formed wass filtrated and washed with water and methanol.

##### Poly(Aniline-Co-Pyrrole) Copolymer

0.02 mol of aniline monomer and 0.02 mol of pyrrole monomer were taken in a round bottomed flask along with 0.06 mol of desired dopants (HCl, H_2_SO_4_, C_6_H_5_-SO_3_-K), and the mixture of reaction was stirred for 30 min at 0–5 C. In another beaker, 13.7 g of APS was dissolved in 30 mL water and stirred in beaker for 30 min at 0–5 °C, which was added to the round bottomed flask containing the reaction mixture while stirring at 0–5 °C; the solid product formed was filtrated and washed with water and methanol.

##### Carbonization/Activation of Prepared Polymers

The carbonization/chemical activation of the prepared polymers was performed taking 2 g of the polymer and mixing it with KOH in weight ratios of 2 and 4, i.e., KOH/Polymer weight ratio of 2 or 4. The mixture was carbonized at 650 °C attained at a ramp rate of 3 °C/min for 2 h using Carbolite furnace under nitrogen atmosphere. The weight of the pyrolysed sample was noted. The pyrolyzed samples were then thoroughly washed with 10 wt % HCl to remove any inorganic salts, and then with distilled water until neutral pH was attained. They were then dried in an oven at 120 °C. The dry weight of the washed sample was noted as weight of N-GOs obtained. The samples prepared were denoted as mentioned in Table 1.

### 2.2. Characterization

Surface area measurements, pore size distribution, and various other surface related parameters were carried out using the BET method based on N_2_ physisorption capacity at 77 K using Gemini VII 2390 V1.03 apparatus (Micromeritics, Norcross, GA, USA) with the instrument operating in single-point and multi-point modes. Prior to analysis, the samples were degassed for 3 h at 150 °C. The activated carbon prepared was also subjected to scanning electron microscopy to find out the surface porosity and morphology.

## 3. Results and Discussion

### 3.1. Textural Properties

**Figure 1:**
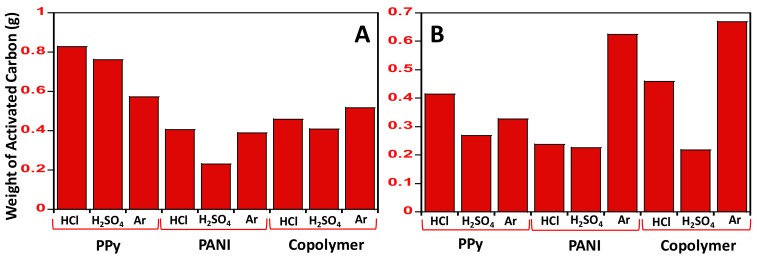
Graphical illustration of amount of activated carbon from pyrolysis of various polymers: (**A**) amount of N-GOs obtained when carbonization and activation were carried using polymer: KOH at a ratio of 1:2; (**B**) amount of N-GOs obtained when carbonization and activation were carried using polymer: KOH in the ratio of 1:4.

The N-GOs obtained after carbonization were weighed, and it was observed that the amount of N-GOs obtained after the acid wash varied based on the polymer used and the dopant added; however, there was no particular pattern observed with regard to it. The amount of polymer used for carbonization was 2 grams, and the highest amount of N-GOs, i.e., about 0.82 g, was obtained when the polypyrrole doped with HCl was carbonized, while the least amount was obtained from the carbonization of copolymer, i.e., Poly(aniline-co-pyrrole) doped with H_2_SO_4_. The amount of carbonaceous material obtained from the various polymers and dopants are illustrated graphically in Figure 1.

**Figure 2:**
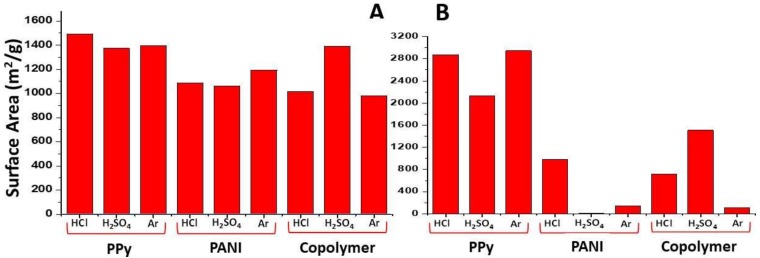
Graphical illustration of surface area of the activated carbon obtained from pyrolysis of various polymers: (**A**) amount of N-GOs obtained when carbonization and activation were carried using polymer: KOH at a ratio of 1:2; (**B**) amount of N-GOs obtained when carbonization and activation were carried using polymer: KOH at a ratio of 1:4.

The surface area analysis revealed that the N-GOs prepared were found to possess surface area varying from 4.75 m^2^/g to ~3000 m^2^/g. It was found that the N-GOs obtained from the carbonization of polypyrrole, polyaniline, and co-polymer, which were mixed with KOH at a ratio of 1:2 (Figure 2A), yielded surface area in the range of ~1063–1556 m^2^/g, while the N-GOs obtained from the carbonization of polypyrrole, polyaniline, and co-polymer, which were mixed with KOH at a ratio of 1:4 (Figure 2B), yielded a wide range of surface area ranging from 4.75 m^2^/g to ~3000 m^2^/g; among these the samples, PPy/HCl-2, PPy/H_2_SO_4_-2, and PPy/Ar-2 yielded samples with surface areas of 2870 m^2^/g, 2134 m^2^/g, and 2943 m^2^/g surface area, respectively. These samples of N-GOs, i.e., PPy/HCl-2, PPy/H_2_SO_4_-2, and PPy/Ar-2, were obtained when the polymer polypyrole doped with HCl, H_2_SO_4_, and C_6_H_5_-SO_3_-K was mixed with KOH during the carbonization step in the ratio 1:4, while the same polymer doped with HCl, H_2_SO_4_, and C_6_H_5_-SO_3_-K was mixed with KOH during the carbonization step in the ratio 1:2. Then, the surface area for the N-GOs obtained was found to be 1491 m^2^/g, 1374 m^2^/g, and 1397 m^2^/g, respectively. This indicates that the amount of KOH mixed with the polymer before carbonization plays an important role in the surface area of the N-GOs obtained. In the case of PPy, the surface area increased up to 64% upon employing KOH in the ratio 1:4 from 1:2; however, in the N-GOs obtained from PANI and Co-poly, the surface area decreased drastically. 

**Figure 3:**
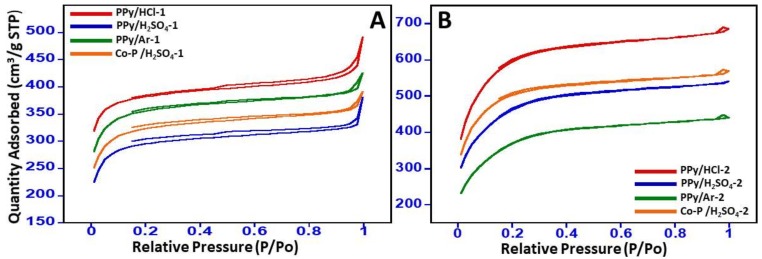
Adsorption/desorption isotherms obtained for (**A**) N-GOs obtained when carbonization and activation were carried using polymer: KOH at a ratio of 1:2; (**B**) N-GOs obtained when carbonization and activation were carried using polymer: KOH at a ratio of 1:4.

The nitrogen adsorption-desorption isotherms for the N-GOs with higher surface areas of more than 1400, i.e., PPy/HCl-1, PPy/H_2_SO_4_-1, PPy/Ar-1, Co-P /H_2_SO_4_-1, PPy/HCl-2, PPy/H_2_SO_4_-2, PPy/Ar-2, and Co-P/H_2_SO_4_-2, were selected for further textural and surface morphological evaluations. The N_2_ adsorption/desorption isotherms were obtained and plotted in Figure 3. The different polymeric precursors, and the amount of KOH and dopants employed for the synthesis were found to have an effect on the textural and surface morphological of the N-GOs obtained, which was ascertained from the different isotherms obtained. The N_2_ adsorption/desorption isotherms curves obtained were found to increase in the low relative pressure, i.e., p/p_o_ < 0.2, indicating the presence of pore structures; the isotherm was found to match to Type I adsorption curves, indicating the presence of mono layers.

However, in the case of N-GOs obtained by employing KOH at a ratio of 1:4, as the relative pressure increases the knee of the isotherm becomes more open than the one obtained in the case of N-GOs obtained by employing KOH at a ratio of 1:2, and a regular increase was observed, indicating the increase in the amount of nitrogen adsorbed with the increase in relative pressure, signifying the formation of mesopores with Type IV isotherm. Hence, it can be said that the N-GOs that were obtained display a combination of the Type I and Type IV isotherms, signifying the formation of microporous and mesoporous N-GOs. Furthermore, the occurrence of hysteresis loop of the H4 type suggests that the pores are random with irregular structures.

### 3.2. Morphological and Microscopic Analysis

**Figure 4:**
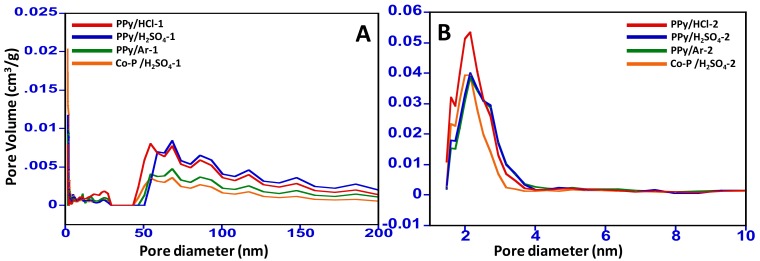
Pore size distribution for (**A**) N-GOs obtained when carbonization and activation were carried using polymer: KOH at a ratio of 1:2 and (**B**) N-GOs obtained when carbonization and activation were carried using polymer: KOH at a ratio of 1:4.

The pore size distribution study was carried out using the BET and the data obtained is presented graphically in Figure 4. Figure 4A shows the microstructure of the inside of N-GOs prepared from the polymer substrates employing 1:2 KOH in the pre-carbonization step, which indicates that the porous structure obtained ranges from 50–200 nm in diameter, with volume ranging from <0.005–0.01 cm^3^/g. However, when the N-GOs prepared from the polymer substrates employing 1:4 KOH in the pre-carbonization step were evaluated, they were found to possess diameter ranging from 1–3.5 nm, while their pore volume ranged from 0.04–0.05 cm^3^/g. From the values obtained, it is clear that the KOH treatment pre-carbonization plays an important role in the surface morphology and porosity of the N-GOs obtained, with the KOH at a ratio of 1:2; the pore diameter ranges from 50–200 nm, and the pore volume denotes that the pores formed are wide and shallow, while with the 1:4 KOH ratio, the pores appear to be long cylindrical capillaries with pore diameter ranging from 1–3.5 nm (Figure 4B).

**Figure 5:**
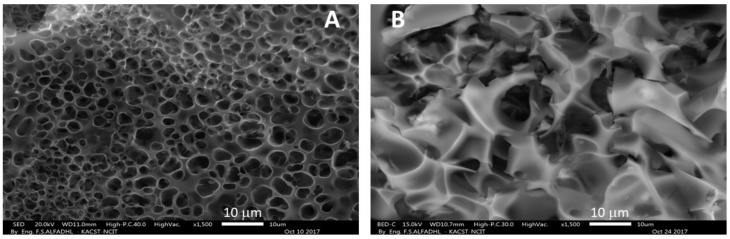
SEM micrograms obtained for N-GOs (**A**) PPy/Ar-1 (**B**) PPy/Ar-2.

**Figure 6:**
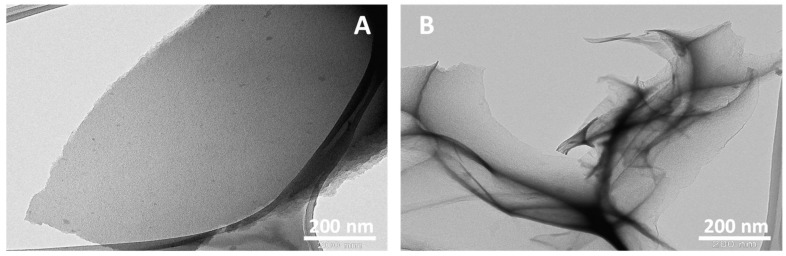
TEM micrograms obtained for graphene sheets-like structure of (**A**) PPy/Ar-1 (**B**) PPy/Ar-2.

The SEM analysis of the obtained N-GOs was carried out to understand the surface morphology, while the TEM analysis helps one understand the formation of sheet-like structure as desired. The SEM and TEM images that were obtained are given in Figure 5 and Figure 6, respectively.

The SEM micrograms obtained indicated that a porous structure was well-formed with rigid borders in case of N-GOs formed from the PPy/Ar-1 obtained when polymer: KOH was at a ratio of 1:2, which is in accordance with the data obtained from the pore size distribution graph obtained as given in Figure 5A. However, in case of the N-GOs formed from the PPy/Ar-2 obtained when polymer: KOH was at a ratio of 1:4, the surface appears to be rugged, and porosity is not very evident, as shown in Figure 5B. Hence, it can be said that the pore size and pore size distribution data of N-GOs is in good agreement with that of SEM images.

The TEM analysis of the samples revealed that a sheet-like structure was formed that resembled the exfoliated graphene sheets. However, from the images it is evident that the N-GOs formed from the PPy/Ar-1 obtained when polymer: KOH was at a ratio of 1:2 appear to be thicker than the ones obtained when the polymer: KOH was at a ratio of 1:4, i.e., PPy/Ar-2, which indicates the activation process, effects the thickness of the sheet formed. The porous structure obtained from the TEM images yielded that it is in the range of 1–3 nm (Figure 6A,B).

### 3.3. XRD Spectral Analysis

**Figure 7:**
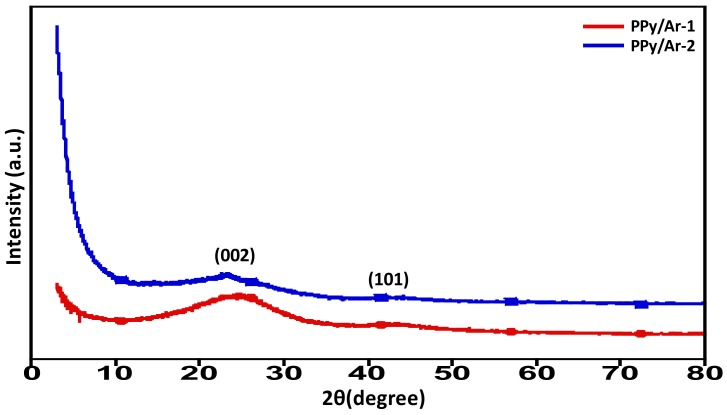
XRD diffractograms obtained for N-GOs PPy/Ar-1 and PPy/Ar-2.

The composition of amorphous carbon is generally made of organized graphite-like microcrystals, non-organized carbon, and single-reticular-plane carbon [33]. The N-GOs PPy/Ar-1 and PPy/Ar-2 that were prepared by different activation methods and were subjected to XRD analysis to confirm the composition and the diffraction patterns obtained are shown in Figure 7. The absence of sharp peaks along with very broad diffraction peaks reveals that the N-GOs formed are amorphous in nature. The two obvious diffraction peaks represent, respectively, the diffraction-characteristic peaks of the microcrystalline (002) and (10l) crystal face of the turbostratic graphite structure of the PPy/Ar-1 and PPy/Ar-2. The slight difference in the diffraction pattern between the two N-GOs PPy/Ar-1 and PPy/Ar-2 can be attributed to the extent of disorderliness prevailing in the graphitic structure of the N-GOs prepared. The varying intensity of the diffraction spectrum suggests that the difference between the surface of the pores in the PPy/Ar-1 are more occupied with the dopants employed, while PPy/Ar-2 appears to be more naked and exposed due to the employment of higher amount of KOH during the activation process [34].

### 3.4. X-ray Photoelectron Spectroscopy (XPS)

**Figure 8:**
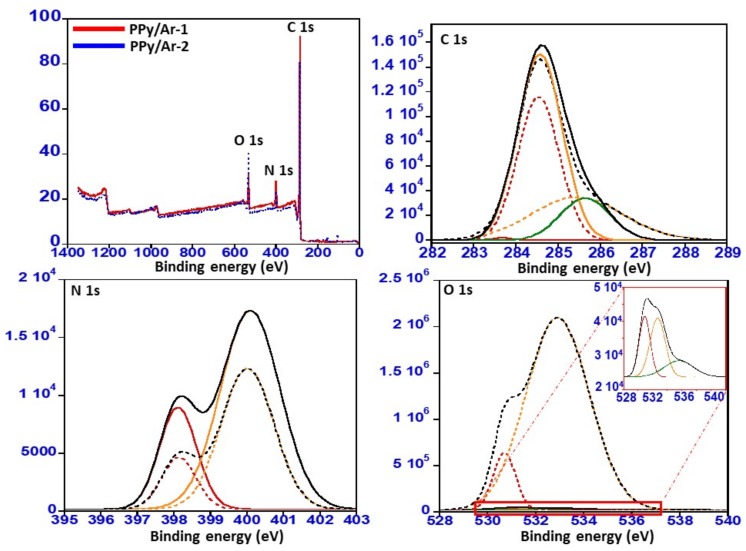
XPS spectrum obtained for N-GOs PPy/Ar-1 (thick line) and PPy/Ar-2 (dotted line).

X-ray photoelectron spectroscopy (XPS) was also conducted to study the chemical compositions of the N-GOs, i.e., PPy/Ar-1 and PPy/Ar-2 were prepared by employing the polymeric precursors, and the results are shown in Figure 8. From the results obtained, it is found that the N-GOs PPy/Ar-1 and PPy/Ar-2 obtained are found to be mainly composed of C, N, and O elements observed in the survey spectrum. The C 1s spectrum of PPy/Ar-1 (thick line) and PPy/Ar-2 (dotted line) yielded a broad peak at 284 eV, which can be attributed to the graphitic carbon and C–N [35]. As for N 1s spectrum of PPy/Ar-1 (thick line) and PPy/Ar-2 (dotted line), it yielded a peak that can be separated into two peaks with binding energy of 398.1 eV and 400.02 eV. The major peak located at 400.02 eV indicates the presence of pyrrolic-N (398–402 eV), while the peak at 398.1 can be assigned to pyridinic-N (397.1–399.3 eV). However, the intensities in the peaks vary, indicating the varying presence of the pyrrolic-N and pyridinic-N, which indicates that the percentage of presence of pyrrolic-N and pyridinic-N is found to be greater in PPy/Ar-1 (thick line) than in PPy/Ar-2 (dotted line) [36,37]. The high-resolution O 1s spectra (Figure 6) in case of PPy/Ar-1 (thick line) reveal the presence of three peaks corresponding to C=O groups (530.8 eV), C–OH, and/or C–O–C groups (532.6 eV), and chemisorbed oxygen and/or water (535.6 eV), while the PPy/Ar-2 (dotted line) yielded two peaks corresponding to C=O groups (530.7 eV), C–OH, and/or C–O–C groups (532.9 eV). A variation in the intensities of the O 1s peaks obtained can be observed in this case, just like in the N 1s spectra. Moreover, the percentage composition calculated revealed that PPy/Ar-1 possesses more N atoms on the surface, while the percentage of O atoms is greater in the PPy/Ar-2 sample. The elemental composition calculated from the XPS spectras of PPy/Ar-1 and PPy/Ar-2 is given in Table 2.

### 3.5. Thermal Stability 

**Figure 9:**
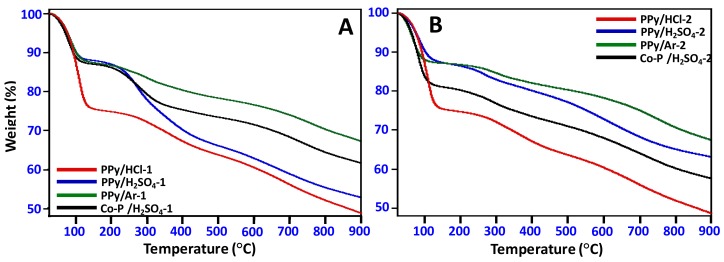
TGA thermograms obtained for (**A**) N-GOs obtained when carbonization and activation were carried using polymer: KOH in the ratio of 1:2; (**B**) N-GOs obtained when carbonization and activation were carried using polymer: KOH in the ratio of 1:4.

The thermal stability of the synthesized N-GOs was evaluated by employing thermal analysis by heating the samples in N_2_ atmosphere from 25 °C to 900 °C, and it is found that different samples displayed differing thermal stability (Figure 9). The best thermal stability is displayed by PPy/Ar-1 and PPy/Ar-2, which displays 32.8% and 32.4% weight loss, respectively, while PPy/HCl displays least thermal stability with a weight loss of ~52%.

### 3.6. CO_2_ Adsorption Properties

**Figure 10:**
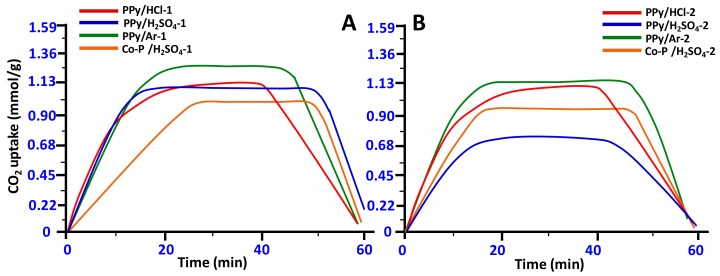
CO_2_ adsorption isotherms measured by TGA-DSC for (**A**) amount of N-GOs obtained when carbonization and activation were carried using polymer: KOH at a ratio of 1:2 and (**B**) amount of N-GOs obtained when carbonization and activation were carried using polymer: KOH at a ratio of 1:4.

The preliminary CO_2_ adsorption-desorption behavior measured at 50 °C and 1.0 atm for the N-GOs samples prepared is shown in Figure 7. Firstly, the N-GOs tested were activated at 120 °C by He flow for 20 min, then cooled down to 50 °C. CO_2_ was then passed over until no further weight gain was observed and a complete adsorption–desorption cycle was completed. Each N-GO was found to gradually adsorb CO_2_ over the first 25 min, which then continued at a slower rate until equilibrium was apparently achieved, which lasted for 60 min for all samples. A graphical representation of the CO_2_ capture pattern exhibited by the various N-GOs is displayed in Figure 10.

It is observed that other than the polymeric precursor and the dopant employed, the activation process plays an important role in the CO_2_ adsorption performance of the N-GOs. Among the various polymeric precursors employed, the N-GOs obtained from the PPy yielded best surface area and pore size distribution. The N-GOs obtained from PPy precursor display the best CO_2_ adsorption efficiency, while the ones obtained from employing the other N-GOs from Co-PPy-PANI also display optimum CO_2_ adsorption capability. Among the N-GOs from PPy, the ones doped with HCl and H_2_SO_4_ display a lower CO_2_ adsorption capability than the ones obtained from C_6_H_5_-SO_3_-K-doped, indicating the role played by the dopant. The N-GOs obtained with varying ratios of KOH effected the CO_2_ adsorption performance of the N-GOs obtained. In case of N-GOs obtained from PPy, when the activation is carried out employing KOH in the ratio 1:2, the N-GOs obtained are found to adsorb CO_2_ better than those of the N-GOs obtained when KOH was used with the ratio of 1:4. However, the adsorption pattern obtained is found to be contrary to the surface area of the N-GOs, which is found to be higher in case of N-GOs obtained employing KOH in the ratio 1:4 than those obtained in case of 1:2. Hence, the adsorption and capture of CO_2_ can be attributed to the porosity of the N-GOs obtained, which in case of polymer: KOH in the ratio of 1:2 was found to possess a wide range of porosity (i.e., 50–200 nm), while in the case of polymer: KOH in the ratio of 1:4, it was found to be narrow (i.e., 1–3.5 nm). The activation process with higher amount of KOH renders the pore more naked and exposed, which reduces the adsorption of CO_2_. Apart from the pore morphology, it is also observed that polymeric precursor and the dopant also play an important role.

The heat of adsorption integrated from the TGA-DSC heat flow curves provides information about the interaction of the CO_2_ molecules and the active sites in the N-GOs. The heat of adsorption is found to be in the range of 10–96 kJ/mol, which indicates that the N-GOs obtained displayed varying interactions between the CO_2_ molecules and the active sites in the N-GOs, leading to the fluctuating adsorption of the CO_2_. Among the N-GOs prepared, the PPy/Ar-1 yielded the highest heat of adsorption, i.e., 96.04 kJ/mol, which is higher and more stable (chemical adsorption) than the heat of adsorption for PPy/Ar-2 (94.13 kJ/mol). 

In case of N-GOs, PPy/Ar-1 was found to possess a pore size of 50–200 nm, while PPy/Ar-2 was found to possess a narrow pore size of 1–3.5 nm. PPy/Ar-1 and PPy/Ar-2 possess pyrrolic-N and pyridinic-N along with C=O groups, C–OH, and/or C–O–C groups. However, the percentage composition varies in the N-GOs prepared, which can be attributed to the difference in CO_2_ adsorption. Of the N-GOs prepared, PPy/Ar-1 possesses a higher percentage of pyrrolic-N and pyridinic-N and wider pore size that could possibly enhance the interaction of the CO_2_, which is indicated by a high heat of adsorption of 96.04 kJ/mol, which greatly promotes CO_2_ adsorption. However, PPy/Ar-2 displays lower CO_2_ adsorption, which can be attributed to the narrow pore size of 1–3.5 nm and the higher percentage of O on the surface of the graphene-like sheets, which could probably hinder the interaction of CO_2_ molecule with the pyrrolic-N and pyridinic-N, which is evident by the lower heat of adsorption 94.13 kJ/mol.

A series of absorbent materials that were previously studied and reported in literature for their adsorption capacities is compiled in the Table 3. When the saturated CO_2_ adsorption capacity of the as-prepared N-GOs is compared to previously reported adsorbent materials, it was found that the as-prepared N-GOs performed better than a few absorbent materials like Cu-propyl ethylenediamine-silica and propyl ethylenediamine-silica composites, while many of the adsorbent materials such as carbonized porous aromatic framework, activated carbon, and mesoporous carbon were found to be better than the as-prepared N-GOs.

## 4. Conclusions

In conclusion, polymeric precursors such as polypyrrole (PPy), polyaniline (PANI), and copolymer (PPy-PANI), along with various dopants such as HCl, H_2_SO_4_, and C_6_H_5_-SO_3_-K, were employed for the preparation of N-doped graphene oxide (N-GOs). Among the polymeric precursors employed, the polypyrrole precursor doped with C_6_H_5_-SO_3_-K yielded the desired N-GOs. The porosity and surface area varied upon the varying use of KOH. The N-doped graphene (NDG) obtained from the use of 1:2, polymer: KOH, i.e., PPy/Ar-1, yielded a porosity in the range of 50–150 nm, while the surface area was found to be 1400 m^2^/g; however, when 1:4, polymer: KOH, i.e., PPy/Ar-2, the porosity was found in the range of 1–3.5 nm, while the surface area was found to be ~3000 m^2^/g. Among the N-GOs prepared, the N-GOs obtained from employing PPy doped with C_6_H_5_-SO_3_-K was found to display the best adsorption property. The N-GOs obtained from PPy doped with C_6_H_5_-SO_3_-K activated by employing KOH at a ratio of 1:2, polymer: KOH, i.e., PPy/Ar-1, yielded a 1.3 mmol/g adsorption of CO_2_; however, when activated with a ratio of 1:4, polymer: KOH, i.e., PPy/Ar-2, yielded a 1.2 mmol/g adsorption of CO_2_. Upon comparison of the obtained adsorption CO_2_ values with the previously reported ones, it was found that the CO_2_ adsorption values obtained were for the as-prepared material, i.e., N-GOs were slightly lower than some of the materials employed earlier, which suggests that these materials can be studied further by fine tuning them to enhance their adsorption properties. This study also revealed a facile synthesis of nitrogen-doped graphene oxide and can be extended to various other polymers, which can be obtained from recyclable material and can be useful for tackling the two environmental issues of the recycling of polymeric waste and air pollution. 

## Figures and Tables

**Table 1:** List of N-GOs samples prepared and denotation method employed in the manuscript.

Polymers	Polymer:KOH					
	1:2 ^3^			1:4 ^3^		
	HCl ^2^	H_2_SO_4_^2^	C_6_H_5_-SO_3_-K ^2^	HCl ^2^	H_2_SO_4_^2^	C_6_H_5_-SO_3_-K ^2^
ppy ^1^	PPy/HCl-1	PPy/H_2_SO_4_-1	PPy/Ar-1	PPy/HCl-2	PPy/H_2_SO_4_-2	PPy/Ar-2
PANI ^1^	PANI/HCl-1	PANI/H_2_SO_4_-1	PANI/Ar-1	PANI/HCl-2	PANI/H_2_SO_4_-2	PANI/Ar-2
Copolymer ^1^	Co-P/HCl-1	Co-P/H_2_SO_4_-1	Co-P/Ar-1	Co-P/HCl-2	Co-P/H_2_SO_4_-2	Co-P/Ar-2

^1.^ Polymer used to prepare the nitrogen-doped graphene oxide sheets; ^2.^ Dopant used when polymer was prepared; ^3.^ Ratio of KOH used along with polymer before carbonization.

**Table 2:** Elemental composition of N-GOs PPy/Ar-1 and PPy/Ar-2.

N-GOs	Elements		
	C	N	O
PPy/Ar-1	80.80	8.08	11.11
PPy/Ar-2	80.80	5.05	14.14

**Table 3:** CO_2_ adsorption capacity comparison of various porous materials with N-GOs PPy/Ar-1 and PPy/Ar-2.

Materials	Capacity (mmol g^−1^)	References
Carbonized porous aromatic framework (PAF)	4.5	[38]
Activated carbon-phloroglucinol-500 °C	4.37	[39]
Microporous carbon ultrafine fibers	2.92	[40]
N-containing porous carbon monoliths	2.9	[41]
Porous carbon nanosheets	2.88	[42]
Alkali-modified activated Carbon	2.46	[43]
Mesoporous carbons	2.27	[44]
Isoreticular zeolitic imidazolate frameworks	2.2	[45]
Mesoporous carbons	2.14	[44]
Commercially activated carbons including BPL, Maxsorb, and Norit R1	<2.00	[46]
Soft-templated mesoporous carbons	1.49	[47]
KOH-activated graphite nanofibers	1.35	[48]
PPy/Ar-1	1.28	This work
PPy/Ar-2	1.18	This work
Cu-propyl ethylenediamine-silica composites	0.58	[49]
Propyl ethylenediamine-silica composites	0.45	[49]
